# Observation of Dirac state in half-Heusler material YPtBi

**DOI:** 10.1038/s41598-020-69284-5

**Published:** 2020-07-23

**Authors:** M. Mofazzel Hosen, Gyanendra Dhakal, Klauss Dimitri, Hongchul Choi, Firoza Kabir, Christopher Sims, Orest Pavlosiuk, Piotr Wiśniewski, Tomasz Durakiewicz, Jian-Xin Zhu, Dariusz Kaczorowski, Madhab Neupane

**Affiliations:** 1 Department of Physics, University of Central Florida, Orlando, FL 32816 USA; 2 Theoretical Division, Los Alamos National Laboratory, Los Alamos, NM 87545 USA; 3 Institute of Low Temperature and Structure Research, Polish Academy of Sciences, 50-950 Wrocław, Poland; 4 Idaho National Laboratory, Idaho Falls, ID 83415 USA; 5 Institute of Physics, Maria Curie - Sklodowska University, 20-031 Lublin, Poland; 6 Center for Integrated Nanotechnologies, Los Alamos National Laboratory, Los Alamos, NM 87545 USA

**Keywords:** Condensed-matter physics, Electronic properties and materials, Topological matter

## Abstract

The prediction of non-trivial topological electronic states in half-Heusler compounds makes these materials good candidates for discovering new physics and devices as half-Heusler phases harbour a variety of electronic ground states, including superconductivity, antiferromagnetism, and heavy-fermion behaviour. Here, we report a systematic studies of electronic properties of a superconducting half-Heusler compound YPtBi, in its normal state, investigated using angle-resolved photoemission spectroscopy. Our data reveal the presence of a Dirac state at the $\Gamma$ point of the Brillouin zone at 500 meV below the Fermi level. We observe the presence of multiple Fermi surface pockets, including two concentric hexagonal and six half-oval shaped pockets at the $\Gamma$ and K points of the Brillouin zone, respectively. Furthermore, our measurements show Rashba-split bands and multiple surface states crossing the Fermi level, this is also supported by the first-principles calculations. Our findings of a Dirac state in YPtBi contribute to the establishing of half-Heusler compounds as a potential platform for novel topological phases.

## Introduction

Topological quantum materials with non-trivial electronic band structures have gained intense research interest due to the possibility of exploiting exotic new physics such as Majorana fermions and the quantum spin Hall effect, as well as for their broad potential applications in quantum computing and spintronics^1–3^. These further lead to the realization and discovery of numerous topologically non-trivial states such as topological insulators (TI), topological Kondo insulators, topological crystalline insulators and topological semimetals, in various material families^4–17^. Interestingly, ternary half-Heusler compounds have been theoretically predicted to provide a platform for realizing non-trivial states in their electronic structure as a result of their innate characteristic to obtain optimized parameters of topological order and topological phase transitions via tunable band gap and diverse spin-orbit coupling (SOC)^18–22^. Furthermore, the half-Heusler compounds containing lanthanide elements with strongly correlated *f*-electrons already have various ground states such as antiferromagnetism and superconductivity^23,24^. Therefore, the presence of *f*-electrons in these systems makes them promising platforms to study the correlated phenomena.

Despite the fact that a decade has already passed since the theoretical prediction of topologically non-trivial states in half-Heusler compounds, there are only a few experimental studies reporting evidences of such states^25–27^. Large and negative longitudinal magnetoresistance has been reported as a signature of the chiral magnetic anomaly in GdPtBi^25^. Recent angle-resolved photoemission spectroscopy (ARPES) studies of LnPtBi (Ln = Lu,Y)^26^ and LuPtSb^27^ have reported the observations of topological surface states. However, the assignment of topological surface state, as well as proposed origin of those states do not agree with each other. In contrast to those two reports, another ARPES study of several half-Heusler phases LnPtBi (Ln = Lu, Dy, Gd)^28^ has not found direct evidence of topologically non-trivial electronic states in these materials. Therefore, it is important to undertake new research efforts in order to conclude the nature of electronic structure of half-Heusler phases.

In this work, we report the detailed electronic structure study of YPtBi using ARPES and first-principles calculations. Our systematic study of electronic structure reveals the hexagonal Fermi surface along with the presence of multiple Fermi pockets. Particularly, we find multiple Fermi surface pockets such as hexagonal and oval pockets around the zone centre and the K points of the Brillouin zone (BZ), respectively. Interestingly, our data reveal a Dirac state at the $\Gamma$ point of the BZ where the Dirac point lies at 500 meV below the Fermi level, which is further supported by our first-principles calculations. Moreover, we report Rashba-splitting in the vicinity of the Fermi surface at the M point of the BZ. We also report the presence of multiple surface states in this system. Our findings bolster the evidence for the existence of exotic states within the half-Heusler family. Furthermore, observation of topologically non-trivial electronic states together with the previously reported superconductivity in YPtBi^29–32^, makes this material a possible platform for further investigations of coexistence of these two phenomena.

## Results

**Figure 1:**
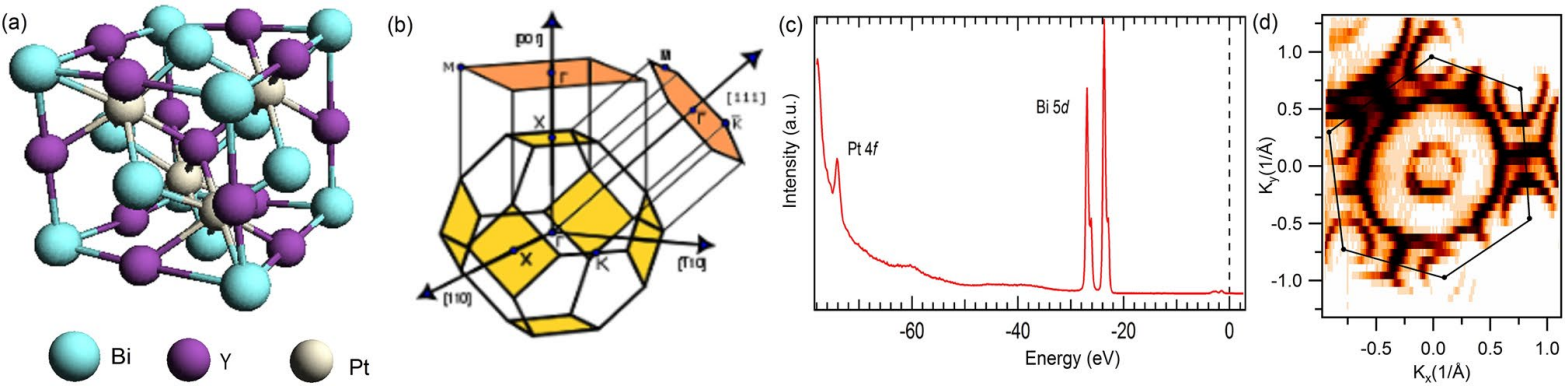
Crystal structure and sample characterization of YPtBi. (**a**) The unit cell of YPtBi compound. (**b**) The bulk Brillouin zone with surface projections along the [001] and [111] directions. High symmetry points are marked in the plot. (**c**) Core level photoemission spectrum. Sharp peaks of Pt 4*f* and Bi 5*d* are observed. (**d**) Spectroscopically measured Fermi surface of YPtBi. Black hexagon represents the first Brillouin zone. Observation of hexagonal Brillouin zone confirms that the cleavage plane of the crystal is (111).

Half-Heusler ternary compounds crystallize with space group $F \bar{4}3m$ lacking inversion symmetry^33^. Figure 1a shows the unit cell of YPtBi. The sample normally cleaves along either the (111) or (001) plane. Figure 1b shows the schematic bulk BZ with surface projections on the (001) and (111) planes. High symmetry points are also marked in the figure, where $\Gamma$ is the centre of the BZ, K is defined as the each corner of hexagon and M is the midpoint of two successive corner points. Figure 1c represents the spectroscopic core level measurement of YPtBi with sharp peaks of Bi 5*d* and Pt 4*f* at around 26.9 eV and 74 eV, respectively. This confirms that the sample of high quality was used for our measurements. Furthermore, larger spectral weight of Bi 5*d* indicates the Bi termination. An ARPES measured hexagonal-shaped Fermi surface of YPtBi is shown in Fig. 1d which further confirms that the cleavage plane of the crystal is (111). The slight distortion in the Fermi surface is due to the small misalignment of the analyser corresponds to the normal emission of photoelectrons from sample.

**Figure 2:**
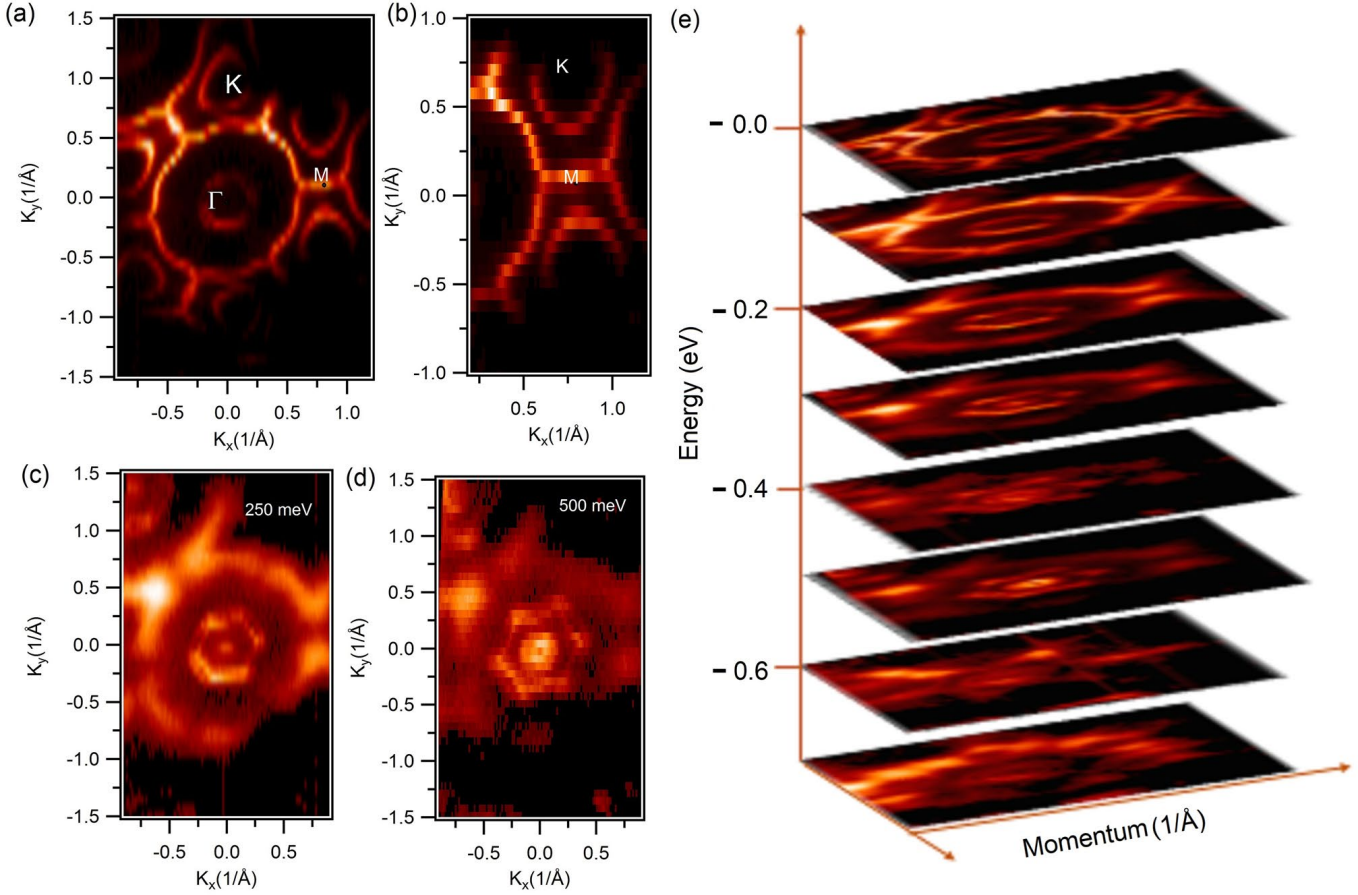
Fermi surface map of YPtBi. (**a**) Fermi surface map at a photon energy of 60 eV. High symmetry points are marked in the plot. (**b**) Zoomed in plot of Fermi surface along the K–M–K direction. (**c**,**d**) Constant energy contour plots at binding energies of 250 meV and 500 meV, respectively. (**e**) Constant energy contour plots stacked in order to show the form of the bands (zero energy corresponds to the Fermi level).

**Figure 3:**
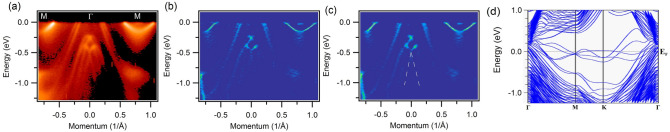
Observation of Dirac state in YPtBi. (**a**) Measured dispersion map along the M–$\Gamma$–M direction at a photon energy of 60 eV. (**b**,**c**) Second derivative plot of (**a**) using the curvature method. White dashed line denoting Dirac state in figure (**c**) serves as a guide for the eyes. (**d**) Theoretical energy dispersion map along the $\Gamma$–M–K–$\Gamma$ high symmetry direction.

Experimental data presented in Figs. 2 and 3 unveil the detailed electronic structure of YPtBi. Figure 2a shows the Fermi surface map revealing the presence of multiple Fermi pockets including two circular-like Fermi pockets around the $\Gamma$ point and six half oval-shaped pockets around the K points of the BZ. Figure 2b presents the zoomed in plot along the K–M–K high symmetry direction and clearly shows the complex half oval-like Fermi pockets. Figure 2c shows the constant energy contour plot at the binding energy of 250 meV, where the circular-like Fermi pocket at the Fermi surface around zone centre point evolves into a twin hexagonal shape. This feature is a consequence of the possible spin-splitting, commonly seen on the surface of the non-centrosymmetric metallic compounds. Furthermore, a small circular feature evolves at the zone centre point as a result of metallic band crossings. Figure 2d shows a constant energy contour in the vicinity of the Dirac point (binding energy $\sim$ 500 meV) in which we observe the circular-shaped feature splitting into two concentric circles. In order to reveal the nature of the Fermi pockets, constant energy contour plots at various binding energies are taken and stacked into an energy versus momentum plot in Fig. 2e. By moving towards the higher binding energy, we observe that the circular-like pocket at the zone centre increases in size and resembles a perfect hexagonal shape. This confirms both the hole-like and hexagonal nature of the pocket. Additionally, the oval-like shape around the K point shrinks into a point and a small circular feature evolves at the zone centre around 250 meV. This confirms the electron-like nature of the Fermi pockets at the K point of the BZ. At the Fermi level we observe a line-like feature crossing M points and connecting the two consecutive BZs as well as separating the two oval-like pockets. However, as we move towards higher binding energies it is observed that the line gap increases and evolves into two distinct segments breaking the outer circle around the $\Gamma$ point. The special outer circle located at around 100 meV below the Fermi level could potentially represent another pair of Kramers points^28^.

**Figure 4:**
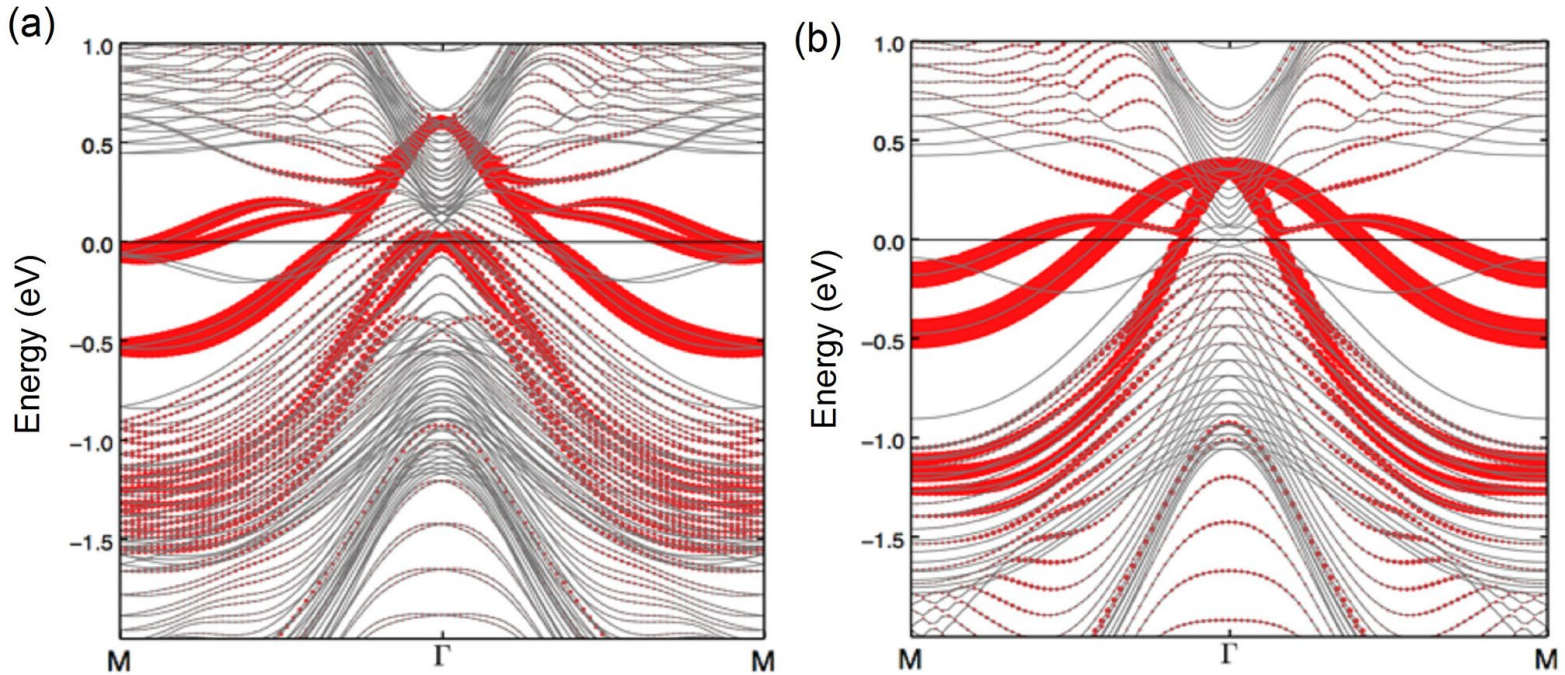
First-principles calculations of Bi-terminated surface state of YPtBi. Electronic band structure of YPtBi along the M–$\Gamma$–M direction (**a**) with inclusion of spin-orbit coupling (SOC) and (**b**) without inclusion of SOC. The red highlight indicates the surface states. The red dots represent the presence of the surface state (Bi atoms at the surface in the slab geometry). The size of red dots shows how much the surface atom is contained in each eigenstate in the band structure. In other words, the larger red circle indicates the more surface atom character contained in the given quasiparticle state.

In order to reveal the nature of the bands, we present in Fig. 3 the dispersion map along the M–$\Gamma$–M direction in which we observe hole-like bands and electron-like bands crossing the Fermi level near the $\Gamma$ point and near the M point, respectively. Interestingly, we observe a Dirac state at the $\Gamma$ point at 500 meV below the Fermi level. Furthermore, we observe another surface state at around 250 meV below the Fermi level. Figure 3b,c presents the second derivative plot using curvature method^34^, where the Dirac point is clearly visible. In order to reveal the origin of the bands, slab calculations were performed which are presented in Fig. 3d. Here, it is clearly observed that the electron-like bands cross the Fermi level. Furthermore, we observe the Rashba-split states in the vicinity of the Fermi level around the M point. The surface calculations (see Fig. 4a) confirm the surface nature of Rashba-split states and the existence of electron-like band at the M-point.

Here, we discuss the results of our first-principles calculations which are presented in Fig. 4. Figure 4a shows the results of calculations of electronic structure for the Bi-terminated surface, with the inclusion of spin-orbit coupling, along the M–$\Gamma$–M direction. The red highlighted bands represent the surface bands. The calculations agree well with our experimental results which show the Dirac point located at around 500 meV below the Fermi level. Furthermore, it shows the Rashba-split band and the electron-like band around the M point. The energy location of the other surface state at the $\Gamma$ point is slightly mismatched with our experimentally observed energy location. Since these bands are the metallic surface state defined within the 2D surface, the position of the surface state might be controlled by impurity potential at the surface. This type of energy mismatching has also been reported earlier^26^. The relative energy shift ($\sim$ 300 meV) between ARPES data and predicted band calculations has been previously reported and is potentially due to the charge imbalance of the cleaved surface (see Ref.^31^). Moreover, electron-like band along the $\Gamma$-M direction is confirmed as surface-originated. Figure 4b displays the first-principles calculations of electronic structure for the Bi-terminated surface without the inclusion of SOC, which does not show a Dirac-like state. We conclude that the linear Dirac bands originate from the spin-orbit coupling arising from heavy Bi atom. The possibility of being Rashba split state or quantum subbands can be ruled out. If it is merely another pair of Rashba split, one would expect two counter propagating states, which is not observed in both our experimental and calculated band structure. Furthermore, such a large Rashba splitting with required Rashaba parameter value $\alpha _R\sim \!2.9\,\mathrm {eV\AA }$ is not expected just by the two-dimensional electronic confinement within the surface band-bending or by the potential gradient arising from the band bending near the Fermi surface.

## Discussion

Considering the experimental and theoretical study of half-Heulser compound YPtBi, both confer the same information regarding the location of the Dirac points at around 500 meV below the Fermi level at the $\Gamma$ point. At this high symmetry point, we notice that the Dirac state originates from the surface which is the result of the strong SOC. Our data qualitatively agree with the study of Liu et al.^26^, however, we do not observe any intrinsic global band gap in YPtBi, which is essential for material to be a topological insulator. This is in accord with the results of our previous studies of specific heat and electrical resistivity, which indicate the semimetallic character of YPtBi^32^. Therefore, we attribute such linearly dispersive band to a Dirac state instead of topological surface state. Moreover, our experimental measurements as well as calculations reveal possible Rashba-split states in the vicinity of Fermi surface at the M point due to an antisymmetric spin-orbit coupling created by the electric field gradient as a consequence of non-centrosymmetric crystal structure.

## Conclusions

In conclusion, we have performed ARPES measurements on the ternary superconducting half-Heusler compound YPtBi in its normal state. Our data reveal the presence of multiple Fermi pockets at the Fermi surface of YPtBi. Furthermore, we directly observe the Dirac state at 500 meV below the Fermi level at the $\Gamma$ point of the BZ. We further observe multiple surface states in our Fermi surface maps. Our first-principles calculations reveal a Rashba-split feature at the M point. We believe that results of our study will stimulate the research interest in investigation of other compounds from half-Heusler family which are possible topological materials.

## Methods

The single crystals of YPtBi were grown from Bi flux as described in Ref.^32^. The chemical composition of YPtBi was checked by energy-dispersive X-ray analysis using a FEI scanning electron microscope equipped with an EDAX Genesis XM4 spectrometer. Homogeneous single-phase materials with their stoichiometry close to equiatomic were observed. Crystal structure of our sample was studied by means of X-ray diffraction on powdered single crystals using an X’pert Pro PAN analytical diffractometer with Cu-K$\alpha$ radiation. It was confirmed that YPtBi crystallises in a space group $F\bar{4}3m$ with lattice parameter $a = 6.65(1) \mathrm{\AA }$, which is in a perfect agreement with previously reported data^29^. The synchrotron based experiments were performed at the ALS BL 10.0.1 equipped with R4000 hemispherical electron analyser at temperature of 15 K. For the synchrotron measurement the energy resolution was set to better than 20 meV and the angular resolution was set to better than $0.2^{\circ }$. The electronic structure calculations were carried out using the full-potential linearised augmented plane wave (FP-LAPW) method implemented in the WIEN2k package^35^, and the PBEsol was used as the exchange-correlation functional^36^. In order to simulate surface effects along the (111) surface, we constructed the hexagonal unit cell of 3 formula units with c-axis along (111) surface. Then we built the $1 \times 1 \times 6$ supercell for the (111) surface, with a vacuum thickness of 20 Å. The spin-orbit coupling was considered with the perturbation in the electronic structure calculations.

## Data Availability

The datasets analysed during the current study are available from the corresponding author on reasonable request.
